# Influence of seasonality on the physicochemical properties of *Moringa oleifera* Lam. Seed oil and their oleochemical potential

**DOI:** 10.1016/j.fochms.2021.100068

**Published:** 2021-12-27

**Authors:** Flávia Michelle Silva Wiltshire, Alessandro de França Santos, Lavínia Kelly Barros Silva, Lays Carvalho de Almeida, Lisiane dos Santos Freitas, Alvaro Silva Lima, Alini Tinoco Fricks, Cláudio Dariva, Cleide Mara Faria Soares

**Affiliations:** a Universidade Tiradentes, Av. Murilo Dantas, 300, Farolândia, Aracaju, SE 49032-490, Brazil; b Instituto de Tecnologia e Pesquisa, Av. Murilo Dantas, 300, Prédio do ITP, Farolândia, Aracaju, SE 49032-490, Brazil; c Departamento de Química, Universidade Federal de Sergipe, Av. Marechal Rondon, s/n Jardim Rosa Elze, 49100-000 São Cristóvão, SE, Brazil; d Departamento de Análises Bromatológicas, Faculdade de Farmácia, Universidade Federal da Bahia, Av. Barão de Jeremoabo s/n, 40170-115 Salvador, BA, Brazil

**Keywords:** CDS, Crude Oil–Dry Season Extract, RDS, Refined Oil–Dry Season Extract, DSR, Dried Seed–Rainy Season Extract, RSR, Raw Seed–Rainy Season Extract, ANOVA, Analysis of variance, HPLC, High-performance liquid chromatography, GC–MS, Gas chromatography–mass spectrometry, FTIR, Fourier transform infrared spectroscopy, *Moringa oleifera*, Oil, Seasonality, Hexane (PubChem CID: 8058), Isopropanol (PubChem CID: 3776), Sodium hydroxide (PubChem CID: 14798), Monoolein (PubChem CID: 5283468), Diolein (PubChem CID: 6505653), Triolein (PubChem CID: 12313712)

## Abstract

The effect of abiotic factors, such as weather and climate can alter the properties of vegetable oils. In this work, the effects of the refining process and pre-drying of Moringa oleifera Lam. seeds collected in the dry and rainy seasons (seasonality) were evaluated on the characteristics of the extracted oils. The refined and crude dry season oils had lower acidity and moisture content than the dry and raw rainy season oils. Oleic acid (C18:1) showed the highest concentration in the different Moringa oils studied. The results suggest that dry season oils are more suitable for application as feedstocks in the cosmetic, food, medicinal and pharmaceutical industries. Furthermore, refining proved to be efficient in removing free fatty acids and in lightening the oil.

## Highlights

• Moringa seeds-dry season provided oils with reduced acidity by at least 7 times.
• Refined oil-dry season (RDS) had undetectable peroxide index.
• Moringa oils-dry season showed higher triacylglycerol content.
• The fatty acid profiles of moringa oils (rainy and dry seasons) were similar.
• RDS is a promising input for the food, medicine and fuel industries.

## Graphical abstract

**Figure:**
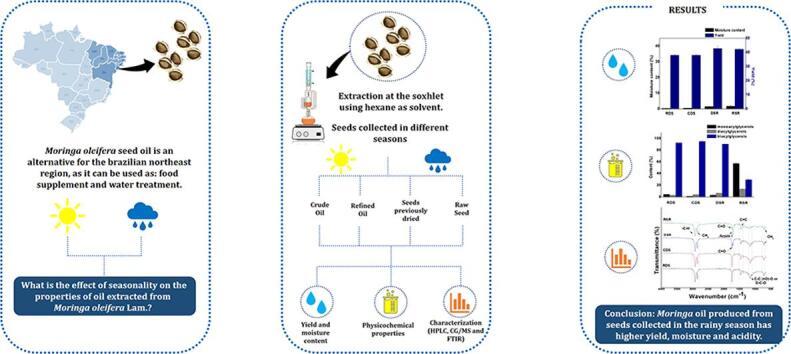

## 1 Introduction

*Moringa oleifera* Lam. is a plant native to northwest India. It is fast growing, has good tolerance to arid soils and is easily adaptable to different climates as such in the Brazil. Currently, its distribution is cosmopolitan, occurring mainly in tropical and subtropical regions (Ayerza, 2019). In Brazil, its dissemination occurs mainly in the northeast region because of its adaptation to arid and semi-arid climates. Its planting is an alternative for this Brazilian region, as the species are a good and safe source of food supplements and can be used in food and as an additive to diseases prevention (Barbosa et al., 2020, Rodrigues et al., 2016, Bezerra et al., 2004). *Moringa* oil has been used in industry for the manufacture of food and biolubricants and has the potential for the production of biodiesel (Liu et al., 2018).

The oil extracted from *Moringa* seeds is rich in monounsaturated fatty acids, with greater than 70% oleic acid and 4.2% linoleic acid and presents high oxidative stability, being similar to olive oil (Ayerza, 2019). The chemical composition of the oil, with high nutritional values associate with possible benefits to human health, such as reducing the risk of cardiovascular diseases and lowering blood sugar levels, make the *Moringa* oil a nutraceutical food (Seifu and Teketay, 2020, Zhong et al., 2018). In addition, *Moringa* esters have the potential for use in lubricants or as ecological additives (Moreira et al., 2020).

The use of vegetable oils deserves to be highlighted, as they are biodegradable, of low toxicity and flammability and possess high indices of viscosity and lubrication, these being recognised as ecological inputs. The oil extracted from the seed of *M. oleifera* Lam. Ben or Behen oil, is a promising new source of vegetable oil (Moreira et al., 2020, Barbosa et al., 2019).

Several factors can affect the quality and quantity of oils from a vegetable source and influencing the yield and chemical composition. Genetic, climatic, seasonal and/or agronomic variables may influence the characteristics of the oil (Raposo et al., 2018). However, further studies are necessary in order to determine factors that affect *Moringa* seeds and consequently the yield and chemical composition of the oil. (Osman and Abohassan, 2012). In this context, the harvest is an important aspect when identifying the ideal moment to obtain a vegetable oil with specific characteristics, mainly in a continental country like Brazil.

The objective of the present work was to present the effect of seasonality on the properties of oil extracted from *M. oleifera* Lam. grown in northeastern Brazil and to determine its influence of the refining of the oils produced.

## 2 Material and Methods

### 2.1 Harvesting, extraction and purification

*Moringa* seeds were collected from plants grown in the Tiradentes University fields (Farolândia Campus) located in Aracaju city, Sergipe/Brazil (10° 58′ 07.8″S 37° 03′ 28.9″ W). For the seasonality study, samples were collected during the dry and rainy seasons. The dry period occurs between the months of August and March and the rainy period from April to July (INMET, 2020). The selected mature seeds are part of the studied population and the selection choice was random, that is there is no preference or intentional choice of any seeds in particular, but of the sample (Barbosa et al., 2020, Rodrigues et al., 2016, Pereira et al., 2016).

Seeds were collected and separated manually from the husk. Then, they were crushed in a blender (Mondial, Conceição do Jacuípe, BA, Brazil) and classified in a series of Tyler sieves, keeping the particle size greater than 60 mesh. Then, 50 g of the sample was added into a soxhlet extractor (Nova Ética, Vargem Grande do Sul, SP, Brazil) for oil extraction in a reflux system at 60 °C for 4 h using 200 mL of n-hexane. The solvent was then separated from the oil by vacuum evaporation. The extraction yield (%) was defined according to Equation (1) (Bhutada et al., 2016).

(1) $$Oil\;yield\left(\%\right)=\frac{M_{1}}{M_{2}}\times 100$$

where M_1_ is the mass of *Moringa* seed oil extracted (g) and M_2_ is the mass of total material processed (g)

To obtain the Refined Oil–Dry Season (RDS) and Crude Oil–Dry Season (CDS) extracts, the samples were degummed. Degumming was accomplished according to the methodology of Pereira et al. (2015): deionised water 20% (w/w) was added to the oil mass and mixed at 500 rpm for 50 min. The oil fraction was separated from the water by centrifugation (5000 rpm for 15 min). The degummed oil was neutralized by the addition of 32% (w/v) of NaOH 3 N and the oil washed three times with cold deionized water (1:1 ratio). The mixture was stirred (1000 rpm) for 30 min, with subsequent centrifugation to investigate the possible presence of an aqueous phase in the oil. The washed oil was heated to 100 °C for 60 min with intermittent agitation every 5 min. The rainy season oils did not undergo the refining process due to the potential of gum formation. However, a sample of rainy season seeds was dried (105 °C for 5 h) from which it was possible to obtain Dried Seed–Rainy Season (DSR) and Raw Seed–Rainy Season (RSR) oil extracts. All reagents were purchased from Neon Comercial. (Suzano, SP, Brazil).

### 2.2 Oil analysis

Peroxide number, iodine value and free fatty acid contents of the oil extract samples (RDS, CDS, DSR and RSR) were determined according to the Official Methods of Analysis of AOAC International.

### 2.3 Statistical analysis

For all oils, the analysis of yield, moisture content, acidity value, free fatty acid contents, iodine value and peroxide value were performed in triplicate. Analysis of variance (ANOVA) was applied using Tukey’s test at 95% confidence to assess the statistical significance of the effects on the results.

### 2.4 High-performance liquid chromatography

Triglyceride content was determined according to Official Method of Analysis (AOAC, 2016) by high-performance liquid chromatography (HPLC). The LC-20AD equipment, fitted with UV–vis detection at 205 nm and an Allcrom 5uC18(2) 100A capillary column (250 × 4.60 mm), was purchased from Shimadzu (Tokyo, Japan). For chromatographic analysis, samples were dissolved (25 mg mL^−1^) in isopropanol/n-hexane, 5:4 (v/v), HPLC grade, were purchased from Sigma Aldrich Ltd. (St. Louis, MO, USA). Chromatographic conditions were injection volume 5 µL, initial temperature 40 °C, flow rate 1200 mL/min. Mono-olein, diolein, and triolein (Sigma Aldrich Ltd., Center County, PA, USA) were used as internal standards for the identification of triglycerides.

### 2.5 Gas chromatography–mass spectrometry

Gas chromatography–mass spectrometry (CG-MS) analysis was performed to determine the composition of the fatty acids present in the *Moringa* oils. For the analysis, the *Moringa* oil was hydrolysed and then derivatized according to the methodology of Barbosa et al., 2019a. Samples were analysed GC–MS (QP2010 plus; Shimadzu, Tokyo, Japan) equipped with an AOC20i autoinjector (split/splitless mode). A DB-5 column (methyl silicone with 5% phenyl groups - film thickness 60 m × 0.25 mm id × 0.25 μm) was used to perform the chromatographic separation. The initial oven temperature was 50 °C for 2 min and then increased steadily up to 300 °C at 10 °C Min^−1^. The injector temperature was kept at 280 °C and the detector at 300 °C. The injection mode used was split (1:20) using helium (ultra-pure, White Martins SA) at 1.4 mL Min^-1^. GC/MS 2.6 software (Shimadzu) was used for the data analysis, and the compound identification was performed by using the database of the NIST-08 spectral library. The identification was based on the comparison of spectra with those from the NIST and WILEY libraries and by detailed analysis of the MS spectra and the position of the peaks considering the retention times in the chromatogram (Barbosa et al., 2019a).

### 2.6 Fourier transform infrared spectroscopy

The *Moringa* oil extract samples were further analysed by Fourier transform infrared spectroscopy (FTIR) at wavelengths from 500 to 4000 cm^−1^, using BOMEM MB-100 FTIR equipment from Oxford Instruments, Abingdon, Oxfordshire, UK.

## 3 Results and discussion

**Fig. 1:**
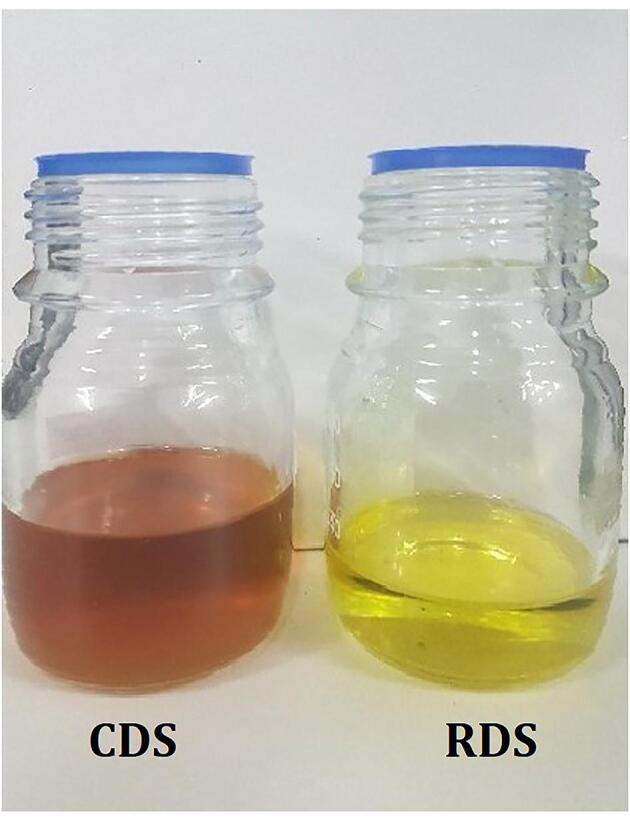
Color change of CDS (Crude Oil–Dry Season Extract) and RDS (Refined Oil–Dry Season Extract) oils before and after the refining process.

The effect of seasonality, refining and drying on oil extraction yield and water content of the extracts (RDS, CDS, DSR and RSR) were evaluated (Table 1). *Moringa* oil produced in the dry season was submitted to the refining process. After refining, a significant reduction (<39%) was observed in the initial volume of the oil. This loss is justified, as the refining process removes impurities (Pereira et al., 2015). It is important to highlight that degumming removes phosphatides and proteins, and the neutralisation removes free fatty acids. In the neutralisation, the free fatty acids react with the aqueous NaOH solution to create soap, which is removed from the oil during centrifugation. Also, changes of appearance (colour) were observed: from the amber-yellow of the CDS to the corn-yellow for refined RDS (Fig. 1). According to Tsaknis (1998), after the degumming process a light-yellow oil is obtained at room temperature, as corroborated in our results.

**Table 1:** Physicochemical analysis of *M. oleifera* Lam. oils.

**Properties**	**RDS**	**CDS**	**DSR**	**RSR**
**Acidity value (mg_KOH_^-1^g_oil_)**	1.82 ± 0.01^a^	2.34 ± 0.09^a^	14.37 ± 0.46^b^	36.71 ± 0.63^c^
**Free fatty acids (mg_KOH_^-1^g_oil_)**	0.91 ± 0.01^a^	1.15 ± 0.09^a^	7.18 ± 0.46^b^	18.35 ± 0.62^c^
**Iodine value (gl_2_100g^-1^_oil_)**	62.14 ± 5.87^a^	69.72 ± 1.32^a^	69.67 ± 0.48^a^	68.45 ± 0.45ª
**Peroxide value (meqO_2_ Kg^-1^_oil_)**	0.00 ± 0.00^a^	4.90 ± 0.32^b^	3.86 ± 0.13^c^	3.78 ± 0.10^c^
**Yield (%)**	34.24 ± 0.14^a^	34.68 ± 0.19^a^	38.92 ± 1.60^b^	38.00 ± 0.22^b^
**Moisture content (%)**	0.32 ± 0.04^a^	0.48 ± 0.03^a^	1.46 ± 0.20^b^	1.82 ± 0.15^b^

Means with different letters on the same line indicate a significant difference at the 95% confidence level by Tukey's test.

RDS: Refined Oil–Dry Season Extract; CDS: Crude Oil–Dry Season Extract; DSR Dried Seed–Rainy Season Extract and RSR Raw Seed–Rainy Season Extract.

The average yields of the dry season oils RDS and CDS were 34.24 ± 0.14% and 34.68 ± 0.19%, respectively, whereas rainy season oils showed an increase in yield (for (38.92 ± 1.60% DSR for 38.00 ± 0.22% for RSR). Similar results have been reported in the literature. The content of degummed *Moringa* oil produced in dry (30,36%) and irrigated (38.37%) regions of Pakistan (the Punjab) varied (Anwar et al., 2006). The moisture content was significantly lower in dry season oils (0.32 ± 0.04% refined and 0.48 ± 0.03% crude) compared to rainy season oils (1.46 ± 0.20% with drying and 1.82 ± 0.15% without drying). The drying process (105° C for 5 h) for seeds collected in the rainy season decreased the water content of DSR; however, these differences were<1%. The higher water content in rainy season oils can contribute to the hydrolysis reaction, consequently promoting an increase in acidity (Table 1). Statistical analysis demonstrated that the yield and moisture content of dry season oils (RDS and CDS) were lower than those of rainy season oils (DSR and RSR).

The physicochemical characteristics of the *Moringa* oil extract (RDS, CDS, DSR and RSR) samples are also are shown in Table 1. The CDS oil showed an acidity index of 2.34 ± 0.09 mg_KOH_^-1^ g_oil_ and that of RDS was 1.82 ± 0.01 mg_KOH_^-1^ g_oil_; there is no significant difference between these values. However, the decrease in acidity is due removal of free fatty acids after the neutralization process (Sánchez-Machado et al., 2015). In the study of Anwar et al. (2006) no significant differences were observed between the acidities of the dried (0.28 ± 0.02%) and irrigated (0.33 ± 0.02%) *Moringa* oil from Punjab, Pakistan. In the present work, a significant increase in acidity was observed for rainy season oils. The acidity of DSR was 14.37 ± 0.4 mg_KOH_^-1^ g_oil_ and that of RSR was 36.71 ± 0.63 mg_KOH_^-1^ g_oil_. The acidity index measures the presence of free fatty acids generated by hydrolysis or by oxidation. The ready adaptation of *Moringa* cultures under different environmental conditions and the chemical composition of the oil extracted from the seeds are decisive factors for choosing the *Moringa* (Moreira et al. 2020). The increase in acidity value in the rainy season oils (DSR and RSR) is probably due to the hydrolysis reactions that can be accelerated by the high moisture content in the seed.

The higher the iodine content, the greater the degree of unsaturated of fats and the more vulnerable they are to oxidative degradation, so higher levels of iodine are related to a greater number of unsaturated double bonds (Goja, 2013). The iodine index varied between 62.14 and 69.72 gl_2_ 100 g^−1^_oil_. There was no statistical significant difference in iodine values among the studied oils and the variation was within the range reported in the literature. (Özcan et al., 2019, Sánchez-Machado et al., 2015, Anwar et al., 2006; Table 1).

In the RDS oil it was not possible to identify the peroxide index with the applied methodology, demonstrating the efficiency of the refining in removing agents that cause oxidation (Pereira et al., 2015, Sánchez-Machado et al., 2015); this differentiated it from the other oils studied. The peroxide value CDS was 4,90 ± 0,32 meqO_2_ kg^–1^_oil_. For DSR and RSR the peroxide indices were 3.86 ± 0.13 and 3.78 ± 0.10 meqO_2_ Kg^-1^_oil_, respectively, the rainy season oils not differing significantly. The values obtained are within the limit for food oils (<10 meqO_2_ Kg^−1^_oil_; Goja, 2013).

The unrefined oils (CDS, DSR and RSR) contain several constituents (moisture, gums, waxes, pigments, antioxidants and free fatty acids), and the refining process removes the most part of these components and makes possible to obtain an odorless oil with greater resistance to oxidation and with more pleasant color and taste. Thus, the refined oil (RDS) has characteristics (Fig. 1 and Table 1) more suitable for its use as an edible oil (Sánchez-Machado et al., 2015).

**Fig. 2:**
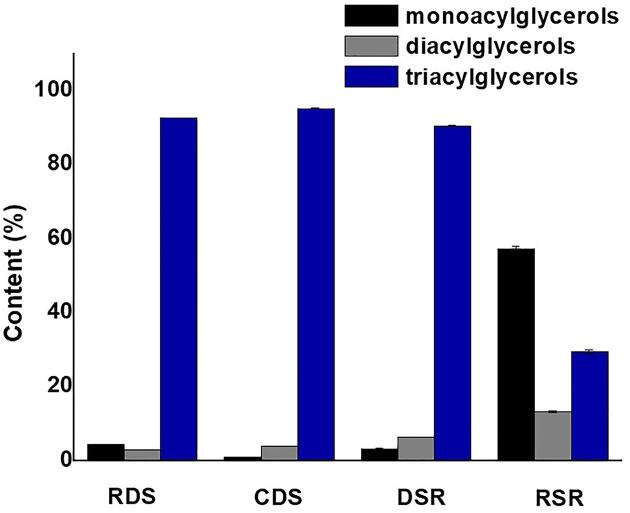
Effect of seasonality and treatment (refining and drying) on profile of mono-di-triglyceride of *Moringa* oils studied: RDS (Refined Oil–Dry Season Extract), CDS (Crude Oil–Dry Season Extract), DSR (Dried Seed–Rainy Season Extract) and RSR (Raw Seed–Rainy Season Extract).

The percentages of monoacylglycerols, diacylglycerols, and triacylglycerols were quantified compared to the mono-olein, diolein and triolein standards, respectively (Fig. 2). The acylglycerol profile of RDS 4.41% monoacylglycerol, 2.88% diacylglycerol and 92.71% triacylglycerol. The CDS contained 0.93% monoacylglycerol, 3.95% diacylglycerol and 95.11% triacylglycerol. The heating of the oil during refining may contribute to the extraction of compounds that cannot be converted into esters, which promotes a reduction in the content of triacylglycerols (Trentini et al., 2020). Similar to other vegetable oils (Correia et al., 2014), triacylglycerides are major components in *Moringa* oil, reaching values around 96% (Zhong et al., 2018). The profile of mono-diglycerides did not differ from those reported in the literature: Jahaniaval et al. (2000) reported that the presence of mono-diglycerides was low for several vegetable oils (4.9% for buckwheat, 7.2% for corn, 8.4% for ricebran, 1, 59% for soybean, 1.37% for sesame, 4.24% for quinoa and 2.94% for cottonseed oils).

In the DSR sample, the triacylglycerol percentage decreased (90.39%). Relating to the effect of seasonality, this observation can be explained by the hydrolysis reaction, considering the higher humidity (1.51 %) in the seeds used in the preparation of this oil extract. In an aqueous environment, tlipase acts by catalysing the hydrolysis of insoluble triacylglycerols to generate free fatty acids, monodiacilglycerols, diacylglycerols, and glycerols (Avelar et al., 2013). One hypothesis is that the presence of water in the *Moringa* seeds possibly allowed the flexibility and molecular folding of lipase, enabling it to adopt its active conformation that favoured the hydrolysis reaction (Caro et al., 2002). The RSR oil extract showed a reduction in triacylglycerol level (29.13%), while the percentages of monoacylglycerol (57.80%) and diacylglycerol (13.05%) were increased compared to other oil extracts. Mono- and diacylglycerols are in greater quantity in crude oil, which can affect physicochemical properties and contribute to the formation of emulsions (Waraho et al., 2012).

The composition of the oil was determined by GC–MS (Table 2). The unsaturated fatty acid fraction was higher than that of the saturated fatty acid fraction in all samples obtained in this study. Fatty acid compositions were 73.12, 74.66, 71.51 and 70.07% unsaturated and 26.88, 25.34, 28.49 and 29.93% saturated in RDS, CDS, DSR and RSR, respectively. Oleic acid was the major fatty acid. Seed oil extracts in Brazil and their oleochemical potential can provide solutions of some medical–biological problems. For example, oils rich in oleic acid have high oxidative stability and fresh consumption is associated with a reducing the risk of cardiovascular diseases. Also, low levels of saturated fatty acids can be seen as a healthy alternative (Gharsallah et al., 2021, Bhutada et al., 2016). The oleic, α-linolenic and linoleic acids together promote reduction in the LDL – low density lipoprotein. In this sense, the Moringa oil is considered a nutraceutical food (Sánchez-Machado et al., 2015). Moreover, when compared to other *Moringa* seed oils extracted with solvents in different regions of the world, the percentage of oleic acid (69.0–73.7%) varied within the range predicted in the literature. No significant differences were found in the amounts of the main fatty acids present in *Moringa* oils. Thus, the constituents verified in present study for *M. oleifera* Lam. seeds oil are in accordance with previous studies by Barbosa et al. (2019a), Oladipo and Betiku et al. (2019) and Abdulkarim et al. (2005). Following the work of Sánchez-Machado et al. (2015), the refining process did not affect the fatty acid profile, so the nutritional value of the oil is not compromised.

**Table 2:** Relative percent composition of fatty acid in *M. oleifera* Lam. oil.

**Fatty acid**	**Structure**	**Determined values**				**Reported values**		
		RDS	CDS	DSR	RSR	Barbosa et al. (2019a)	Oladipo and Betiku (2019)	Abdulkarim et al. (2005)
Palmitic	C16:0	8.77 ± 0.0	9.33 ± 0.7	8.24 ± 0.3	9.11 ± 0.3	8.30 ± 0.3	6.17	7.8 ± 1.25
Palmitoleic	C16:1	ND (*)	ND (*)	ND (*)	ND (*)	ND (*)	1.29	2,2 ± 0.06
Stearic	C18.0	8.04 ± 0.3	7.98 ± 0.9	7.58 ± 0.7	8.48 ± 0.2	5.00 ± 0.2	5.58	7.6 ± 0.05
Oleic	C18:1	72.40 ± 1.6	73.70 ± 0.2	70.59 ± 0.0	69.02 ± 0.5	74.50 ± 0.5	71.31	67.9 ± 0.75
Linoleic	C18:2	0.72 ± 0.0	0.96 ± 0.0	0.92 ± 0.2	1.05 ± 0.1	2.50 ± 0.2	0.61	1.1 ± 0.10
Arachidic	C20:0	2.88 ± 0.7	1.69 ± 0.0	3.66 ± 0.3	3.24 ± 0.3	3.00 ± 0.1	3.87	4,0 ± 0.06
Behenic or docosanoic	C22:0	6.28 ± 0.5	5.67 ± 0.2	7.61 ± 0.1	7.62 ± 0.1	2.00 ± 0.2	8.12	6.2 ± 0.50
Lignoceric	C24:0	0.91 ± 0.2	0.67 ± 0.2	1.40 ± 0.1	1.48 ± 0.0	3.00 ± 0.1	ND (*)	1.3 ± 0.00

(*) ND – not determined

RDS: Refined Oil–Dry Season Extract; CDS: Crude Oil–Dry Season Extract; DSR Dried Seed–Rainy Season Extract and RSR Raw Seed–Rainy Season. Extract.

**Fig. 3:**
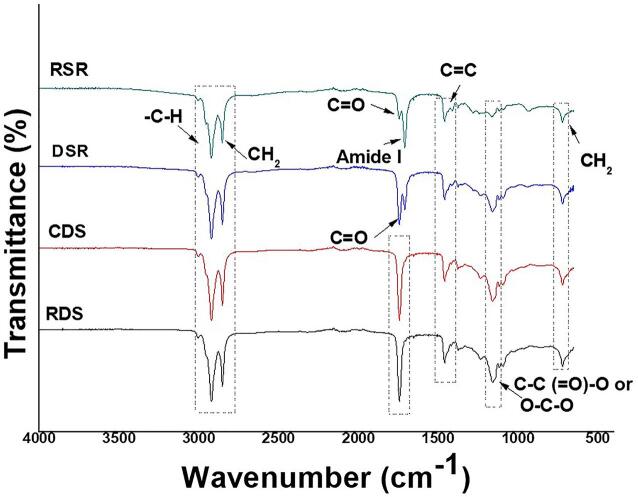
FTIR analysis of *Moringa oleifera* Lam. The analyzed oils were: RDS (Refined Oil–Dry Season Extract), CDS (Crude Oil–Dry Season Extract), DSR (Dried Seed–Rainy Season Extract) and RSR (Raw Seed–Rainy Season Extract).

All FTIR spectra exhibited bands characteristic of the fatty acids and lipid components (Fig. 3): extensive bands at 2922 and 2953 cm^−1^, attributed to the symmetrical and asymmetric elongations of C–H (CH_2_); a band between 1744 and 1708 cm^−1^, referred to the ester carbonyl group (C = O; Barbosa et al., 2020, Oladipo and Betiku, 2019); and a band at 1459 cm^−1^ regarding to aromatic C = C bond. The band at 1159 cm^−1^ is attributed to the diacylglycerol esters and represents asymmetric stretches of the C–C(=O), –O and O–C–O bonds. The band at 721 cm^−1^ is due to asymmetric deformation of the CH_2_ group (Moreira et al., 2020, Oladipo e Betiku 2019, Bhutada et al. 2016). The seasonality effect was verified with the presence of a band at 1700 cm^−1^ in the DSR and RSR oil extracts. Bands between 1700 and 1600 cm^−1^ are related to the α-helix structure in proteins (Barbosa et al., 2019b). These data suggest the presence of proteins in these DSR and RSR extracts of *Moringa* oil. The presence of water in rainy season extracts promoted an increase in free fatty acids due to the hydrolysis reaction (Table 1, Table 2). The water (a weak nucleophile) attacks the ester linkage of triacylglycerols generating mono-diacylglycerols, in addition to glycerol and free fatty acids (Ogunsina et al., 2014) (Fig. 2).

The crude and refined Moringa oils investigated are considered relatively clean-burning, and potentialas renewable fuel. The refined oil has high potential for biodiesel production, but mainly as feedstock for cosmetic, food, medicinal and pharmaceutical industries (Pereira et al., 2016). The physicochemical properties (regarding free fatty acids and peroxide) and fatty acid composition (C18:1) reveal that Moringa seed oil can be a raw material for industrial applications, especially the refined oil (RDS).

Moringa seed oil is rich in several antioxidant compounds with therapeutic properties and, among them, tocopherols (fat-soluble vitamins) stand out. Moringa seed contains α-, γ- and δ-tocopherols, with α-tocopherol (vitamin E) being the most abundant. α-Tocopherol is associated with the prevention of cardiovascular diseases, in addition to preventing lipid oxidation in the body, such as that of polyunsaturated fatty acids and cellular components (membranes and organelles). Thus, Moringa seed oil is a green source of liposoluble vitamins and a potential source of bioproducts of industrial interest (Gharsallah et al., 2021).

## 4 Conclusion

The seasonality effect influenced the yield of *Moringa* oil from *M. oleifera* seeds. The higher moisture content proportionally influenced an increase in the acidity in the rainy season oils (DSR and RSR). In these oils, the presence of water in the seeds may have contributed to the hydrolysis reaction, favouring the higher content of mono- and diacylglycerides in the RSR. The refining process allowed the removal of free fatty acids and made possible to obtain an oil resistant to oxidation and with a better color without affecting the fatty acid profile. Therefore, this work seeks to inform future studies regarding the best harvest season, in Brazil and other countries, with relation to extraction yield and free fatty acid value of oils from *M. oleifera* seeds.

## Declaration of Competing Interest

The authors declare that they have no known competing financial interests or personal relationships that could have appeared to influence the work reported in this paper.
